# Blood transfusion services amidst the COVID-19 pandemic

**DOI:** 10.7189/jogh.11.03053

**Published:** 2021-04-17

**Authors:** Siti Salmah Noordin, Narazah Mohd Yusoff, Faraizah Abdul Karim, Soon Eu Chong

**Affiliations:** 1 Cluster of Regenerative Medicine, Advanced Medical and Dental Institute, Universiti Sains Malaysia, Kepala Batas, Malaysia; 2 Hemophilia Clinic, National Blood Centre, Kuala Lumpur, Malaysia; 3 Hospital Ampang, Ministry of Health Malaysia, Selangor, Malaysia; 4 Department of Anaesthesiology and Intensive Care, School of Medical Sciences, Universiti Sains Malaysia, Kelantan, Malaysia; 5 Hospital USM, Health Campus, USM, Kubang Kerian, Kelantan, Malaysia

The year 2020 marked the beginning of a major change in the way we humans live and interact due to the COVID-19 pandemic [1,2]. The causative agent of this disease is severe acute respiratory syndrome coronavirus 2 (SARS-CoV-2). Due to the relatively mild symptoms in the early phase of infection, a long incubation period and a high infectivity rate, SARS-COV-2 has spread at lightning speed globally. To date, the number of COVID-19 cases has exceeded 120 million globally and continues to rise exponentially with a fatality ratio of 0.5%–1.0%, and this ratio increases with age. Despite headlines on COVID-19 vaccines and treatments, the ability of the virus to mutate leaves many uncertainties particularly with regard to the efficacies of vaccines and treatments.

Healthcare services, including blood transfusion, have been significantly affected. This review aims to discuss the impact and suggest the best practice for transfusion services in this era of new norms (**Figure 1**).

**Figure 1:**
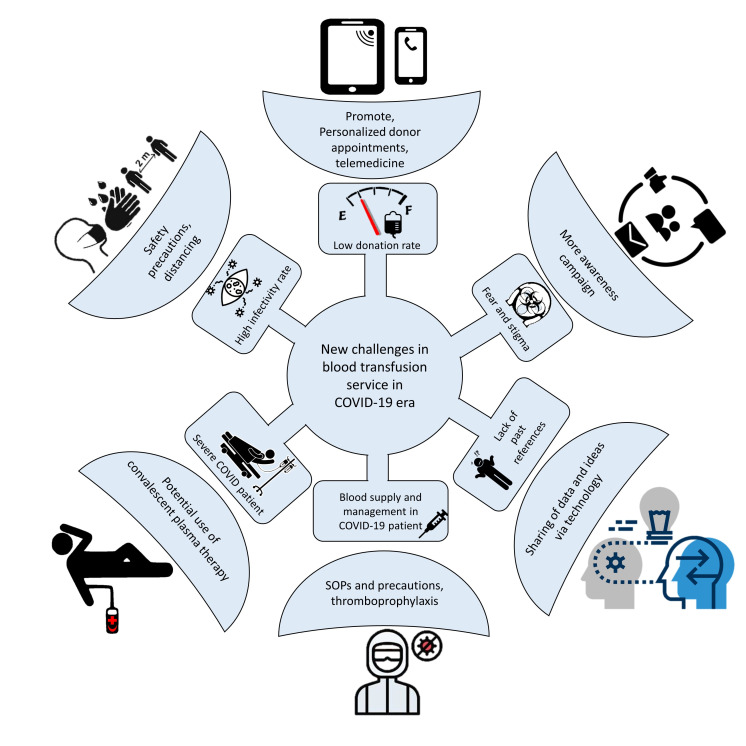
New challenges in blood transfusion services in COVID-19 era and suggested steps to establish. SOP, standard operating procedures.

## DONATION SAFETY

### Blood donors

Blood donations have decreased dramatically by 40% to 67% during this outbreak of COVID-19, particularly in countries where restriction of movement is enforced [3]. Potential blood donors face difficulties to access donation sites and fear of contracting the virus along the way. In addition, most mobile blood drives have been cancelled.

To address this problem, continuous public appeals for donation through both mainstream and social media are crucial coupled with reassurances on safety measures taken throughout the process. Blood centres can actively approach potential blood donors via emails, text messages or phone calls. These telemedicinal approaches can also be used as a first health screening to ensure that only suitable donors turn up for donation and allowing personalised donation slots to be arranged. Transportation may also be provided for donors.

Stringent donation criteria should be implemented. A 14-day deferral from donation should be applied to those who have recovered from COVID-19 and those who have received live-attenuated COVID-19 vaccines.

**Figure:**
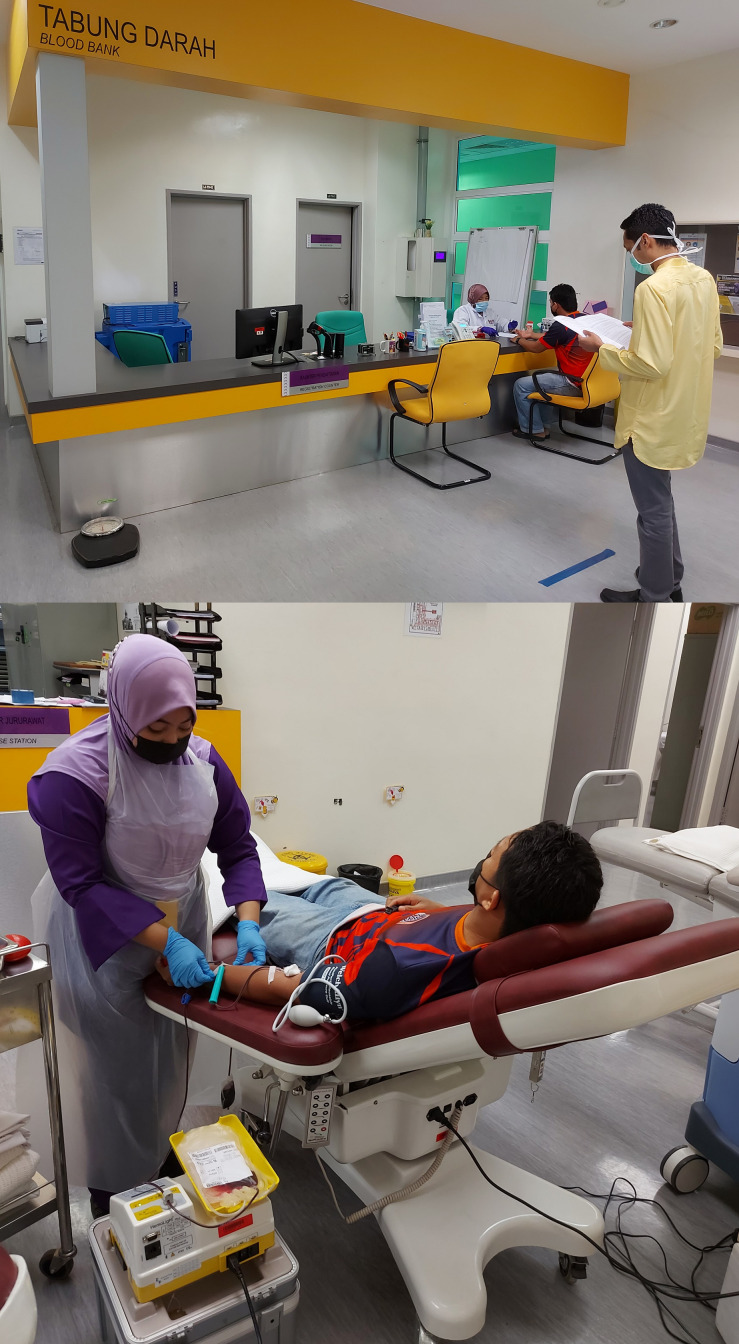
Photo: Malaysian blood bank operating in the COVID-19 pandemic by conforming to the new norms (from Siti Salmah Noordin and Ermarina Kamaruddin’s collection, used with permission).

### Blood collection sites

**Figure 2:**
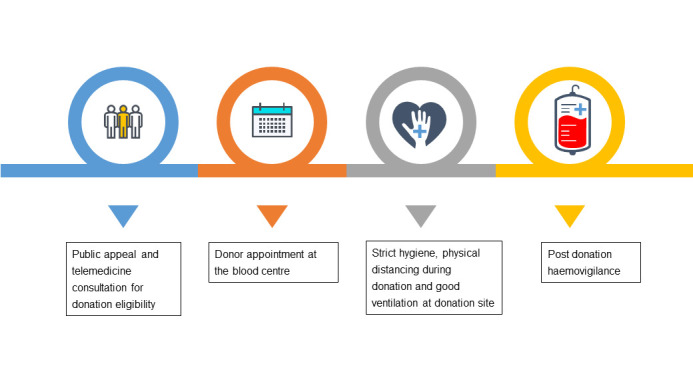
Blood donation activities during COVID-19 pandemic.

Healthcare workers (HCWs) are not spared from being infected by COVID-19. A meta-analysis estimated that the prevalence of SARS-CoV-2 infection among HCWs is about 7%-11% [4]. Thus, HCWs should strictly adhere to infection control practices during this pandemic. (**Figure 2**)

In blood donation areas, chairs need to be rearranged in accordance with physical distancing guidelines. High-touch areas and equipment should be frequently disinfected. The recommendation of ventilating blood donation areas twice a day for not less than 30 minutes each time should also be followed [5].

## BLOOD PRODUCT SAFETY

SARS-CoV-2 is transmitted mainly through respiratory droplets and direct contacts. To date, there has been no reported case of blood transfusion-transmitted COVID-19 even among patients who were transfused with blood from donors that was found to be positive for COVID-19 [6,7]. However, concerns and anxiety still persist among HCWs regarding the handling of COVID-19 blood samples.

Blood product screening for viruses may have a high false-negative rate which questions its usefulness, similar to what happened during the SARS outbreak [8,9]. Nevertheless, pathogen reduction (PR) on blood products using riboflavin and UV (UV) light has been shown to be effective in reducing SARS-CoV-2 infectivity [10].

Other additional strategies to ensure the safety of blood products include prolonged quarantine of RBCs and plasma products for up to 14 days; this recommendation is based on a model estimation study which reported about 65.77% reduction risk of SARS-CoV-2 transmission through blood [11].

Active haemovigilance of blood donors and recipients must be emphasised. Any possibility of COVID-19 transmission via blood transfusion must be properly investigated. The use of information technology is important in the tracing of transfusion chains [12]. If blood donors are suspected or confirmed to have COVID-19, lookbacks and recall procedures should be performed on their donated blood products and their recipients.

## TRANSFUSION TESTING AND BLOOD SUPPLY FOR PATIENTS WITH COVID-19

It has been reported that about 13.4% of hospitalised patients with COVID-19 received blood transfusion, and this number is lower than non-COVID-19 patients. RBCs remain the highest blood product transfused (11.1%) [13]. Handling of blood samples from patients with suspected or confirmed COVID-19 should follow strict laboratory biosafety practices according to the national health recommendations. In view of the low concentration of virus in non-respiratory samples, blood tests can be performed under standard blood-borne pathogen biosafety level (BSL)-2 [14].

All samples and request forms for blood transfusion should be clearly labelled indicating the sample is from a COVID-19 patient. Triple-layer packaging is recommended for transporting the sample to the laboratory. The outer layer of biohazard plastic bag and container should be disinfected prior to transportation. In some centres, UV irradiation is used for disinfection prior to laboratory processing [10,15].

**Figure 3:**
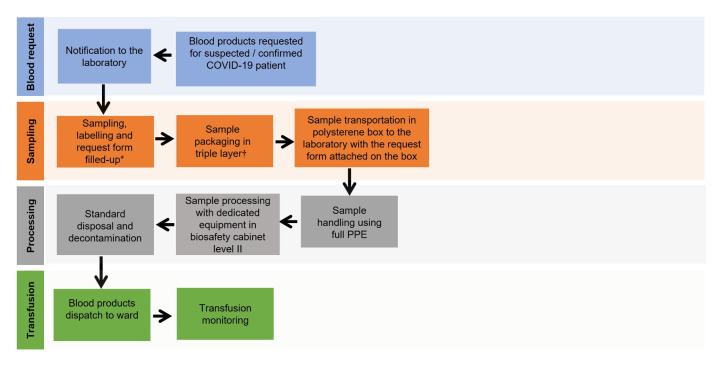
Transfusion process for suspected or confirmed case of COVID-19. PPE, personal protective equipment: disposable gown, gloves, N95 masks, eye goggles or face shields, work caps, shoe covers and plastic aprons. *Both sample tubes and request forms should state ‘COVID-19 sample’. †Triple layer packaging: sample tube seal with paraffin film, wrapping the tube with gauze and tightened it with rubber band (first layer), placement of the tube into two consecutive biohazard bags (second and third layer). Disinfect the outer biohazard bag with 70% alcohol.

Laboratory personnel who handle COVID-19 samples are required to wear full personal protective equipment (PPE) and process the samples in a biosafety cabinet. After each step of centrifugation, the samples should be left for at least 15 minutes to avoid aerosolising of the content. Other steps of pre-transfusion testing can be performed according to standard operating procedures (SOP), with additional precaution on safe handling. After completion of testing and analysis, laboratory equipment should be decontaminated using proper disinfectants, such as ethanol (70%-95%), isopropanol (50%-100%), 0.5% hydrogen peroxide, 0.1% sodium hypochlorite and 0.7% glutaraldehyde (**Figure 3**).

In some blood transfusion services, test procedures such as group, screen and hold (GSH) and crossmatching will not be provided for COVID-19 patients. Should these patients require blood transfusions, uncrossmatched blood group O packed-RBCs, group O platelet or group AB plasma are supplied for transfusion for those who have no history of sensitisation. However, transfusion should be performed with caution because of the risk of non-ABO mediated alloantibody which may lead to delayed transfusion reaction. Nevertheless, shortages of group O RBCs for group O patients should be anticipated if all patients with COVID-19 are routinely supplied through this approach.

## Coagulopathy in patients with severe COVID-19

Among patients with severe COVID-19, cytokine storms and liver injury may predispose them to coagulopathy. Severe cases of COVID-19 may present as pneumonia and sepsis. These conditions may produce hyperactive T lymphocytes, massive release of interleukin-6 and interleukin-1, which contribute to a cytokine storm. These phenomena lead to extensive tissue damage, endothelial injury and release of tissue factors that may promote a widespread thrombin generation, leading to disseminated intravascular coagulopathy (DIC).

The up-regulation of angiotensin-converting enzyme 2 (ACE2) expression in liver tissues caused by compensatory hepatocyte proliferation may also lead to COVID-19-related liver injury [16]. Severe liver tissue injury in turn reduces the production of vitamin K-dependent coagulation factors, protein C and protein S, resulting in coagulopathy.

Interestingly, the abnormal coagulation parameters in severe COVID-19 infection do not often lead to bleeding. Transfusion of blood components should only be reserved for coagulopathic patients with high risk of bleeding or requiring invasive procedures. Replacement of blood products by relying solely on laboratory parameters might worsen the disseminated thrombosis.

In view of the prothrombotic state, prophylactic dose of low-molecular-weight heparin is generally recommended for most of hospitalised patients with COVID-19, irrespective of prothrombin time or activated partial thromboplastin time, unless the platelet counts are below 25 × 10^9^/L or fibrinogen level is below 0.5 g/L. Mechanical thromboprophylaxis should be employed in cases where pharmacological therapy is contraindicated.

## CONVALESCENT PLASMA THERAPY

Thus far, efforts in finding the best treatment for COVID-19 is still ongoing. Convalescent plasma (CP) and hyperimmune immunoglobulin has been used as a potential treatment for patients with COVID-19 who do not respond well to other treatments. These treatments involve passive transfer of antibodies collected from donors who have recovered from prior infection. Therapy with CP provides neutralising antibodies and other proteins which may ameliorate severe inflammatory response through immunomodulation [17]. The use of passive immunisation as a treatment for infection is not a new technology and can be dated back to the 1890s, whereby serum was used to treat diphtheria. For the past two decades, CP has been successfully administered to treat SARS, influenza A and Middle East Respiratory Syndrome.

Several criteria are required for donor recruitment to ensure the safety of CP products. Such criteria include donors with confirmed RT-PCR for SARS-CoV-2, tested negative for SARS-CoV-2 after at least 14 days of symptoms resolution, anti-SARS-Cov-2 neutralising antibody activity titre minimum of 1:40 and tested negative on all the standard microbiology screening tests [18]. Plasma is collected via apheresis with volume of approximately 600 mL, and the process can be performed every two weeks.

At present, no standard dose for CP has been established, but administering 3 mL/kg per dose within two days is generally recommended. Clinical trials showed that the effectiveness of CP and hyperimmune immunoglobulin against COVID-19 are still uncertain [19]. Some trials were discontinued prematurely either due to lack of samples or patient safety issues.

## CONCLUSION

Apart from ensuring the safety of donors, HCWs and patients, encompassing technology in the management of blood transfusion service and the sharing of latest updates are important contributions to the improvement of health care services in this COVID-19 era.
